# Introduction to LJPC issue 9.1

**DOI:** 10.1080/17571472.2016.1274935

**Published:** 2017-01-09

**Authors:** Paul Thomas

Happy New Year everyone.

Issue 9.1 signals a 2017 new development for LJPC as we start to develop collaborating sites to illuminate *community*-*oriented integrated care and health promotion* (COIC) in different contexts. The four papers are written by LJPC editors and have arisen from discussions at the LJPC Board about how to support authors to evaluate COIC, to highlight emerging issues in ways that reveal practical ways forward, and to learn from colleagues in different places about how to work with skill and humanity in complex situations.

• Read: *Collaborating Sites for COIC* if you might like to join a network of thoughtful people from different parts of the world and submit occasional papers about how you are interpreting COIC.
• Read: *The Use of Case Studies to Drive Bottom*-*Up Leadership in COIC* if you want to know how case study methodology can illuminate the complex and constantly changing nature of the ‘real world’.
• Read: *General Practice Nursing: who is cherishing this workforce?* if you want to consider ways to address the present crisis in practice nurse recruitment.
• Read: *Henri de Toulouse*-*Lautrec’s talent* – *and troubles* – *were larger than life* to be reminded that interaction between physical, social and emotional health is a natural feature of everyday life.

